# *Vairimorpha (Nosema) ceranae* can promote *Serratia* development in honeybee gut: an underrated threat for bees?

**DOI:** 10.3389/fcimb.2024.1323157

**Published:** 2024-05-13

**Authors:** Chiara Braglia, Daniele Alberoni, Paula Melisa Garrido, Martin Pablo Porrini, Loredana Baffoni, Dara Scott, Martin Javier Eguaras, Diana Di Gioia, David Mifsud

**Affiliations:** ^1^ Dipartimento di Scienze e Tecnologie Agro-Alimentari (DISTAL), University of Bologna, Bologna, Italy; ^2^ Centro de Investigación en Abejas Sociales (CIAS), Faculty of Exact and Natural Sciences (FCEyN), National University of Mar del Plata (UNMdP), Mar del Plata, Buenos Aires, Argentina; ^3^ Instituto de Investigaciones en Producción Sanidad y Ambiente (IIPROSAM), National Scientific and Technical Research Council (CONICET), UNMdP, Centro Asoc. Simple Scientific research Commission Buenos Aires Province (CIC PBA), Mar del Plata, Buenos Aires, Argentina; ^4^ Advance Science Ltd., Galway, Ireland; ^5^ Institute of Earth Systems, L-Universita ta’ Malta, Msida, Malta

**Keywords:** *Serratia*, nosemosis, *Nosema ceranae*, fumagillin, beneficial bacteria, *Apilactobacillus*

## Abstract

The genus *Serratia* harbors opportunistic pathogenic species, among which *Serratia marcescens* is pathogenic for honeybees although little studied. Recently, virulent strains of *S. marcescens* colonizing the *Varroa destructor* mite’s mouth were found vectored into the honeybee body, leading to septicemia and death. *Serratia* also occurs as an opportunistic pathogen in the honeybee’s gut with a low absolute abundance. The *Serratia* population seems controlled by the host immune system, but its presence may represent a hidden threat, ready to arise when honeybees are weakened by biotic and abiotic stressors. To shed light on the *Serratia* pathogen, this research aims at studying *Serratia*’s development dynamics in the honeybee body and its interactions with the co-occurring fungal pathogen *Vairimorpha ceranae*. Firstly, the degree of pathogenicity and the ability to permeate the gut epithelial barrier of three *Serratia* strains, isolated from honeybees and belonging to different species (*S. marcescens*, *Serratia liquefaciens*, and *Serratia nematodiphila*), were assessed by artificial inoculation of newborn honeybees with different *Serratia* doses (10^4^, 10^6^, and 10^8^ cells/mL). The absolute abundance of *Serratia* in the gut and in the hemocoel was assessed in qPCR with primers targeting the *luxS* gene. Moreover, the absolute abundance of *Serratia* was assessed in the gut of honeybees infected with *V. ceranae* at different development stages and supplied with beneficial microorganisms and fumagillin. Our results showed that all tested *Serratia* strains could pass through the gut epithelial barrier and proliferate in the hemocoel, with *S. marcescens* being the most pathogenic. Moreover, under cage conditions, *Serratia* better proliferates when a *V. ceranae* infection is co-occurring, with a positive and significant correlation. Finally, fumagillin and some of the tested beneficial microorganisms could control both *Serratia* and *Vairimorpha* development. Our findings suggest a correlation between the two pathogens under laboratory conditions, a co-occurring infection that should be taken into consideration by researches when testing antimicrobial compounds active against *V. ceranae*, and the related honeybees survival rate. Moreover, our findings suggest a positive control of *Serratia* by the environmental microorganism *Apilactobacillus kunkeei* in a in vivo model, confirming the potential of this specie as beneficial bacteria for honeybees.

## 1 Introduction

The decline in bee populations and the arising consequences are well-known to the agricultural sector. Climate changes and the widespread use of herbicides reduce the feed availability for pollinators due to a decreased flower production and delay in flowering time (Le Conte and Navajas, 2008; Carpenter et al., 2020). The stress derived from the reduced sources of foraging may find a synergy with other environmental or anthropogenic factors in the agroecosystem (Brandt et al., 2016; Raymann et al., 2017), leading to a further weakening of honeybees. For instance, it was recently demonstrated that the gut microbial community of honeybees can be altered by exposure to herbicides (Motta et al., 2018), insecticides (Alberoni et al., 2021b), and antibiotics (Baffoni et al., 2021), downregulating the expression of host-produced antimicrobial peptides, therefore altering the insect immune system (Motta et al., 2022) and pathogen susceptibility (Li et al., 2017; Castelli et al., 2020).

Among bees, honeybees are the most studied pollinators because of their economic relevance, in terms of not only their pollination services but also their colony products. A great number of honeybee microbial pathogens, whose virulence can be increased by environmental stressors, are known, including *Vairimorpha ceranae* (or syn. *Nosema ceranae*) (Higes et al., 2013) and viruses such as the chronic bee paralysis virus (Martin et al., 2013; Zheng et al., 2015; Ullah et al., 2021). In addition, in recent years, opportunistic bacterial pathogens such as *Hafnia*, *Klebsiella*, *Serratia*, and *Enterobacter* have drawn attention. These bacteria co-exist with their host and normally do not cause a disease, but they can become pathogenic or increase their relative abundance when the host’s defense system is impaired (Brown et al., 2012; Raymann and Moran, 2018) or in honeybees exposed to pesticides (Al Naggar et al., 2022; Al Naggar and Wubet, 2024). For example, some of them produce chitinases, which can seriously damage the exoskeleton of invertebrates or help them under certain conditions (Gooday, 1990). Iverson et al. (1984) found that *Serratia liquefaciens* and *Serratia marcescens* can penetrate in the pupae of *Tetanops myopaeformis*, establishing a symbiotic association that helps the beet root maggot emergence. Conversely, in some crab species, *Serratia* causes serious exoskeleton damage (Baross et al., 1978). However, the role of opportunistic bacteria is poorly investigated despite their potential impact on the host, especially *Serratia*.

The interaction between quality and abundance of feed sources and susceptibility to diseases has been observed in many organisms (Dolezal and Toth, 2018; Dolezal et al., 2019). Although mechanisms involved are not yet well known, it was postulated that pollen and nectar contain proteins, lipids, carbohydrates, and a variety of phytochemicals and micronutrients that may affect immune response. In addition, feed quality has been found to alter the proportion of some core gut microorganisms in honeybees, for both artificial (Alberoni et al., 2021a; Ricigliano et al., 2022) and natural nutrition (Castelli et al., 2020). Consequently, a possible reduction or loss of specific gut microbiome taxa can alter the protective functions of the immune system (Kwong et al., 2017) and potentially induce the expression of pathogenicity traits by opportunistic microorganisms (Raymann et al., 2018; Horak et al., 2020) like *Serratia*.

*Serratia* is a Gram-negative opportunistic environmental pathogen that may colonize the hemolymph of larvae and adult honeybees causing septicemia (Grimont and Grimont, 1978; Burritt et al., 2016). In adults, the bacterium causes a reduction of motility up to paralysis (Burritt et al., 2016), while larvae die and appear whitish, sticky, and smelly (El Sanousi et al., 1987) with a symptomatology like foulbrood. *Serratia* can be present in low amounts as a non-core bacterial species in the honeybee gut microbiota (Raymann et al., 2017, 2018); it is recognized that, when *Serratia* gut strains colonize the hemolymph and body cavity (hemocoel), it becomes highly virulent (Burritt et al., 2016; Raymann et al., 2018) as an opportunistic pathogen. However, a well-performing core gut microbiome is correlated with the production of antimicrobial compounds and host immune stimulation that leads to the control of pathogen proliferation in the gut, as documented for *S. marcescens* (Steele et al., 2017).

Concerning *S. marcescens*, a particularly virulent strain was isolated by Burritt et al. (2016) and named strain *Sicaria* (Ss1). This strain was responsible for atypical winter losses, and it was discovered to colonize the mouthpart of *Varroa destructor*. Moreover, the *Sicaria* strain has proven its virulence when vectored by *V. destructor* and injected directly into the honeybee hemocoel (Burritt et al., 2016). After this first isolation, Raymann et al. (2018) isolated other *S. marcescens* strains showing a similar mode of infection. The presence of *Varroa* mites associated with gut microbiota perturbations appear to be optimal conditions for opportunistic pathogens widespread in adult honeybees (Hubert et al., 2016). Therefore, conditions leading to honeybee stress could be determinant for *Serratia* proliferation and pathogenesis. For this reason, the aim of this research is to establish a possible connection between an immune depressing and tissue damaging honeybee gut parasite such as *V. ceranae* and *Serratia* colonization. In fact, *V. ceranae* is well known to cause severe gut epithelial damages (Martin et al., 2013). In this work, the pathogenicity of three different *Serratia* species isolated in Italy from honeybee gut was assessed together with their ability to colonize hemocoel through the gut epithelial cell wall under laboratory conditions. Moreover, the synergy with the gut pathogen *V. ceranae* was assessed and evaluated after the administration of a feed supplement based on microorganisms, pollen, and the antibiotic fumagillin already tested against *Vairimorpha* (Alberoni et al., 2018; Garrido et al., 2024). In particular, the presence and amount of the co-occurring and opportunistic pathogen *Serratia* were taken into consideration in a sample subset produced in Garrido et al. (2024) and a possible correlation with *V. ceranae* was considered.

## 2 Materials and methods

### 2.1 Isolation of *Serratia* strains and effect of the isolated strains on honeybee survival

The isolation was performed both from the gut content of honeybees and from hive debris. Approximately 15 worker honeybees were collected from an experimental apiary (Valsamoggia, Bologna, Italy) in 2014 and promptly transferred to the laboratory. All bees were sacrificed by freezing, and their gut content was extracted and mixed to obtain 1 g. One gram of colony debris was also collected. Both isolation matrixes were serially diluted in a 0.85% NaCl solution and plated on GYC Agar (50 g/L dextrose, 10 g/L yeast extract, 5 g/L calcium carbonate, and a 10 g/L agar medium with no addition of antibiotics or ethanol). Plates were incubated for 5 days at 30°C under aerobic conditions. Strains were selectively isolated based on morphology, growth rate, and color. Isolates were characterized with a PCR-dependent fingerprinting technique based on the enterobacterial repetitive intergenic consensus (ERIC) sequence. PCR was carried out with primers ERIC-1 (5’-ATGTAAGCTCCTGGGGATTCAC-3’) and ERIC-2 (5’-AAGTAAGTGACTGGGGTGAGCG-3’) as described in Alberoni et al. (2019). Fingerprinting profiles were analyzed with GelCompar II 6.6 (Applied Maths, Kor-trijk, Belgium) using the DICE coefficient and the UPMGA clustering algorithm. DNA amplification of the 16S rRNA gene was performed for samples with an ERIC unique profiles, with primers 27f (5’-AGAGTTTGATCCTGGCTCAG-3’) and 1492r (5’-GGTTACCTTGTTACGACT-3’) according to Alberoni et al. (2019). Amplicons were purified and sent to a commercial sequencing facility (Eurofins MWG, Ebersberg, Germany). Sequence chromatograms were analyzed, manually edited, and classified using BLAST tool from NCBI. The honeybee survival test using the *Serratia* isolated strains was performed according to Garrido et al. (2024), whereas the mortality and diet consumption information are reported in the **Supplementary Materials** of Garrido et al. (2024). Briefly, 50 newly emerged honeybees were caged and fed with *Serratia* inoculum of 1.2 × 10^8^ CFU/mL, 1.2 × 10^6^ CFU/mL, or 1.2 × 10^4^ CFU/mL incorporated in sugar syrup (sucrose:water 1:1 w/v). Each experimental condition was repeated in triplicate and was performed with three different *Serratia* strains belonging to the species *S. liquefaciens*, *S. marcescens*, and *S. nematodiphila*. Dead honeybees were registered and removed daily as well as the amount of feed consumed.

### 2.2 Infection with *Serratia* strains and quantification of *Serratia* in the gut and hemocoel

Two- to three-day-old honeybees were manually collected from a mother colony located in Valsamoggia (Bologna district, Italy, 44°25’54.0”N 11°02’55.2”E). In the laboratory, honeybees were individually infected with 1.13 × 10^4^, 1.13 × 10^6^, and 1.13 × 10^8^ cells of *S. liquefaciens*; 1.8 × 10^4^, 1.8 × 10^6^, and 1.8 × 10^8^ cells of *S. marcescens*; and 2.5 × 10^4^, 2.5 × 10^6^, and 2.5 × 10^8^ cells of *S. nematodiphila*. The prepared bacterial suspensions were administered with sugar syrup (sucrose:water 1:1 w/v) at a final volume of 10 μL/bee. For each *Serratia* strain dilution, 50 honeybees were caged and maintained at 29°C ± 2 and 60% RH. Fresh tap water and sugar syrup were supplied *ad libitum* and replaced every day. Every 2 days, three honeybees per cage were sacrificed and prepared for the DNA extraction: gut was carefully separated from the abdomen, and guts and the fat bodies with hemocoel processed separately although attached to the abdomen. DNA extraction was performed as described in Section 2.5, and *Serratia* was quantified as described in Section 2.5. The experimental conditions and abbreviations are defined in **Table 1**, section 1.

**Table 1:** Summary of cage experiment description.

In cage test				
Acronyms	Short description	Full description of the treatment	Sampling time	Reference article
1. Correlation between Serratia gut infection and septicemia				
**C**	Uninfected control	Sugar syrup^a^	**Every 3 days for 10 days**	**This work**
**M4**	*S. marcescens* AC8	Sugar syrup^a^ + 10^4^ CFU *S. marcescens/*bee		
**M6**	*S. marcescens* AC8	Sugar syrup^a^ + 10^6^ CFU *S. marcescens*/bee		
**M8**	*S. marcescens* AC8	Sugar syrup^a^ + 10^8^ CFU *S. marcescens*/bee		
**L4**	*S. liquefaciens* AC4	Sugar syrup^a^ + 10^4^ CFU *S. liquefaciens*/bee		
**L6**	*S. liquefaciens* AC4	Sugar syrup^a^ + 10^6^ CFU *S. liquefaciens*/bee		
**L8**	*S. liquefaciens* AC4	Sugar syrup^a^ + 10^8^ CFU *S. liquefaciens*/bee		
**N4**	*S. nematodiphila* 1A	Sugar syrup^a^ + 10^4^ CFU *S. nematodiphila*/bee		
**N6**	*S. nematodiphila* 1A	Sugar syrup^a^ + 10^6^ CFU *S. nematodiphila*/bee		
**N8**	*S. nematodiphila* 1A	Sugar syrup^a^ + 10^8^ CFU *S. nematodiphila*/bee		
2. Increasing doses of infective V. ceranae spores				
**C**	Uninfected control	Sugar syrup^a^ + UV- treated beebread^c^	**9 days PE**	Garrido et al. (2024)
**CV1**	*Vairimorpha* 500	Sugar syrup^a^ + 5×10^2^ *V. ceranae* spores/bee + UV-treated beebread^c^		
**CV2**	*Vairimorpha* 5,000	Sugar syrup^a^ + 5×10^3^ *V. ceranae* spores/bee + UV-treated beebread^c^		
**CV3**	*Vairimorpha* 50,000	Sugar syrup^a^ + 5×10^4^ *V. ceranae* spores/bee + UV-treated beebread^c^		
**FV1**	Fumagillin 500	Sugar syrup^a^ + Fumagillin 2.59 mM + 5×10^2^ *V. ceranae* spores/bee + UV-treated beebread^c^		
**FV2**	Fumagillin 5,000	Sugar syrup^a^ + Fumagillin 2.59 mM + 5×10^3^ *V. ceranae* spores/bee + UV-treated beebread^c^		
**FV3**	Fumagillin 50,000	Sugar syrup^a^ + Fumagillin 2.59 mM + 5×10^4^ *V. ceranae* spores/bee + UV-treated beebread^c^		
3. Single bacterial strains and mixture—infection with V. ceranae				
**CV+P**	Infected control	Sugar syrup^a^ + *N. ceranae* ^b^ + UV-treated beebread^c^	**9 days PE**	Garrido et al. (2024)
**AKV+P**	Dan39	Sugar syrup^a^ + 5×10^7^ CFU/mL *A. kunkeii* ^d^ + *V. ceranae* ^b^ + UV-treated beebread^c^		
**LPV+P**	Dan91	Sugar syrup^a^ + 5×10^7^ CFU/mL *L. plantarum* ^d^ + *V. ceranae* ^b^ + UV-treated beebread^c^		
**LJV+P**	Dan92	Sugar syrup^a^ + 5×10^7^ CFU/mL *L. johnsonii* ^d^ + *V. ceranae* ^b^ + UV-treated beebread^c^		
**BAV+P**	DSM 20431	Sugar syrup^a^ + 5×10^7^ CFU/mL *B. asteroides* ^d^ + *V. ceranae* ^b^ + UV-treated beebread^c^		
**BCV+P**	C155	Sugar syrup^a^ + 2.1 × 10^6^ CFU/mL *B. coryneforme* ^d^ + *V. ceranae* ^b^ + UV-treated beebread^c^		
**BIV+P**	DSM 20214	Sugar syrup^a^ + 5×10^7^ CFU/mL *B. indicum* ^d^ + *V. ceranae* ^b^ + UV-treated beebread^c^		
**BV+P**	Bacterial mixture	Sugar syrup^a^ + 10^8^ CFU/mL of each bacterial strain^d^ + *V. ceranae* ^b^ + UV-treated beebread^c^		
**FV+P**	Fumagillin + *V. ceranae* + beebread	Sugar syrup^a^ + UV- treated beebread^c^ + Fumagillin 2.59 mM+ *V. ceranae* ^b^ + UV-treated beebread^c^		

In the table, the different cage experiments, the different dietary treatments, and their relative acronyms are reported; in all of them, the number of copies of Serratia was analyzed. Dose and composition of each administration are reported. **^(a)^** 1:1 sucrose: water (w/v); **^(b)^** inoculation with 5×10^4^ V. ceranae spores/bee; **^(c)^** 2 g of pollen/cage; **^(d)^** cell suspension made in sugar syrup [1:1 sucrose: supernatant (w/v)].

### 2.3 *Vairimorpha ceranae* and microorganisms-based feed additives influence on *Serratia* proliferation

To obtain *V. ceranae* fresh spores necessary to perform the controlled infections, forager honeybees were collected from diseased hives [experimental apiary of Social Bees Research Center (CIAS), Buenos Aires Province, Argentina, 38°10’06”S, 57°38’10”W]. When spores were unavailable, to obtain fresh and infective spores, *V. ceranae* was multiplied by infecting 35 newly emerged and caged honeybees, allowing pathogen proliferation into the gut epithelium and sporulation for 14 days.

To reach honeybee genetic homogeneity, newly emerged honeybees were obtained from brood frames picked from multiple colonies and incubated until honeybee emergence at 32°C and 60% RH. The newly emerged honeybees were maintained under controlled conditions (29°C; 60% RH) for 3 days until individual inoculation with freshly prepared *V. ceranae* spores. Briefly, according to Porrini et al. (2013), each single starved honeybee was put in an infection panel and fed with 10 μL of *V. ceranae* spores mixed with sugar syrup.

In each laboratory experiment, homogeneous cohorts of worker honeybees from healthy hives were used. Groups of 50 newly emerged honeybees were placed into cages (12 cm × 8 cm × 6 cm) per treatment per replicate according to Porrini et al. (2020). They were fed daily with fresh tap water, freshly obtained beebread when it was necessary, and syrup to act as a vehicle for the respective treatments. Individuals were maintained at 29°C ± 2 (40% RH) during the assays.

In all cases, DNA was extracted from the gut content and used for the quantification of *Serratia* as described below. Some DNA samples were stored as part of wider studies according to Braglia et al. (2021) and Garrido et al. (2024), and others were obtained in this study (**Table 1**).

#### 2.3.1 Effect of increasing amounts of *V. ceranae* spores on *Serratia*

The test aimed at determining whether different infective doses of *V. ceranae* affect endogenous *Serratia* proliferation. Honeybees were inoculated with 5 × 10^2^, 5 × 10^3^, and 5 × 10^4^ V*. ceranae* spores according to Garrido et al. (2024). At the same time, the antibiotic fumagillin [F] was also assessed for the indirect control of *Serratia*. The experimental conditions and abbreviations are shown in **Table 1**, section 2.

#### 2.3.2 Bacterial strains in *V. ceranae-*infected honeybees

Three assays were performed exclusively with bacterial strains isolated from honeybee midgut. The first one aimed at comparing the effect of six single bacterial taxa and a mixture of them in the *Serratia* control, in the presence of *V. ceranae* infection. *Bifidobacterium asteroides* DSM20431, *Bifidobacterium coryneforme* C155, *Bifidobacterium indicum* DSM20214, *Apilactobacillus kunkeei* Dan39, *Lactiplantibacillus plantarum* Dan91, and *Lactobacillus johnsonii* Dan92, previously described in Baffoni et al. (2016), were grown according to Alberoni et al. (2019). Incubation conditions were standardized for each strain at 35 ± 2°C for 24 h for *Lactobacillaceae* and 72 h for *Bifidobacteriaceae*. Microbial cultures were centrifuged, and pelleted cells were resuspended in sugar syrup. When a suspension of all these bacteria was used for honeybee feeding, it is referred to as bacterial mixture [B]. This was prepared in sugar syrup suspending grown bacterial cells in order to have 5 × 10^7^ CFU/mL of each bacterial strain except for *B. coryneforme* C155 that showed growth problems, and a concentration of 2.1 × 10^6^ CFU/mL was obtained in the sugar syrup. The six single bacterial strains and their mixture were administered orally to honeybees infected with 5 × 10^4^ V*. ceranae* spores according to Garrido et al. (2024). The experimental conditions and abbreviations are shown in **Table 1**, section 3. The samples obtained were analyzed by qPCR to obtain the number of copies of *Serratia* according to Section 2.5 in *V. ceranae-*positive samples.

### 2.4 Agar well diffusion assay to evaluate *Serratia* sensitivity to fumagillin

The assay was carried out, as described by Cintas et al. (1995), on soft agar inoculated with indicator strains. The indicator strains used were *S. marcescens* AC8, *S. liquefaciens* AC4, and *Serratia nematodiphila* 1A. Fumagillin dicyclohexylamine salt was used at the following concentrations: 2.59 mM, 1.30 mM, 0.648 mM, 0.324 mM, 0.162 mM, and no antibiotic. Fumagillin was added in the wells and the sensitivity was assessed controlling the formation of inhibition halos. The assay was performed in triplicate.

### 2.5 DNA extraction and qPCR reaction

Honeybees for each experimental condition per replicate were sacrificed as described in Garrido et al. (2024), and the guts were individually collected and processed according to Braglia et al. (2021) and Garrido et al. (2024). Briefly, guts were manually crumbled with plastic micro pestles in 200 μL of buffer, and DNA extraction of single honeybee guts was performed with a High Pure PCR template preparation kit (Roche Diagnostic; Buenos Aires, Argentina) following the manufacturer’s protocol. Fluorometric quantification of every sample was performed with a Qubit Flex Fluorometer (Thermo Fisher Scientific), and finally, extracted DNA was stored at −20°C until further analysis. The quorum-sensing *luxS* gene was selected as a molecular marker to perform *Serratia* quantitative PCR. The primers used were as follows: luxS1: 5’-TGCCTGGAAAGCGGCGATGG-3’; luxS2: 5’-CGCCAGCTCGTCGTTGTGGT-3’, specifically designed for *Serratia*’s quorum-sensing autoinducer-2 (AI-2) *luxS* gene by Joyner et al. (2014). The standard curve was prepared according to Baffoni et al (2012, 2016). Briefly, standard curves were constructed using PCR products of the *luxS* gene for the target microbial genera. The PCR products were purified with NucleoSpin^®^ Gel and PCR Clean-up (Macherey-Nagel), quantified with Qubit high sensitivity, converted in total amount of copies per microliter and then serially diluted to obtain standards ranging from 5×10^3^ to 5×10^8^ gene copies.

The rRNA small ribosomal subunit gene was selected to perform *V. ceranae-*specific qPCR with primers Nc841f: 5’-GAGAGAACGGTTTTTTGTTTGAGA-3’ and Nc980r: 5’-ATCCTTTCCTTCCTACACTGATTG-3’ (amplicon size ~140 bp) designed by Huang and Solter (2013). Standard curve was prepared as described above. Reactions were carried out on a StepOne thermal cycler (Applied Biosystems) with the standard two-step PCR method using Fast SYBR Green PCR Master Mix (Life Technologies, Milan, Italy), according to Garrido et al. (2024).

### 2.6 Statistical analysis

The survival/mortality test was carried out by comparing different groups by Log-Rank survival curves and Mann–Whitney tests, respectively, unless otherwise noted, according to Garrido et al. (2024). Data were analyzed with R software (de Micheaux et al., 2013). Shapiro and Levene’s tests were performed for every dataset to test normality and homoscedasticity distribution of data. The dataset was corrected with Cook’s Distance multivariate method only for cage tests, to identify outliers based on regression analysis comparison (Cook, 1979). Datasets were analyzed with ANOVA when data were normal and homoscedastic, while GLM was applied for non-normal homoscedastic data. The Bonferroni *p*-value correction model was used for multiple comparisons in every independent assay. Correlation analyses were performed with the R packages *devtools* and *ggpubr* relaying on Pearson’s correlation test. For the correlation on the second test, CV2 and CV3 were deleted from the analysis since the opposite trend of CV2 and CV3 was caused by the higher *Vairimorpha* inoculum. Finally, boxplots and average plots were generated with *ggpubr* and *ggplot2* packages, and where necessary, they overlapped with Adobe Illustrator. *Serratia* quantification was expressed as *luxS* gene copies/gut.

## 3 Results and discussion

The understanding of honeybees’ pathogen dynamics, in particular the synergies among different pathogens, as well as their interactions with environmental or anthropogenic factors (climate changes, feed resources, and pesticides), is crucial for the beekeeping sector. Knowledge of the synergies between pathogen(s) and stressors may allow the design of target strategies to support honeybees’ health. A previous work (Alberoni et al., 2023) has shown that the administration of a bacterial mixture of different *Lactobacillaceae* and *Bifidobacterium* strains, tested to counteract *N. ceranae*, seemed to reduce the natural proliferation of *Serratia* in the gut. In the same work, when honeybees were not treated with the bacterial mixture, *Serratia* was found to proliferate, together with other gut microorganisms such as *Citrobacter*, *Cosenzaea*, and *Morganella*, and therefore, it was suggested that the selected mixture of microorganisms could control *Serratia* under cage conditions. In the present work we aimed at investigating the impact of different beneficial bacterial and their mixture, on *Serratia*, in case of co-infection with *N. ceranae.*

### 3.1 Virulence of isolated *Serratia* strains in honeybees

**Figure 1:**
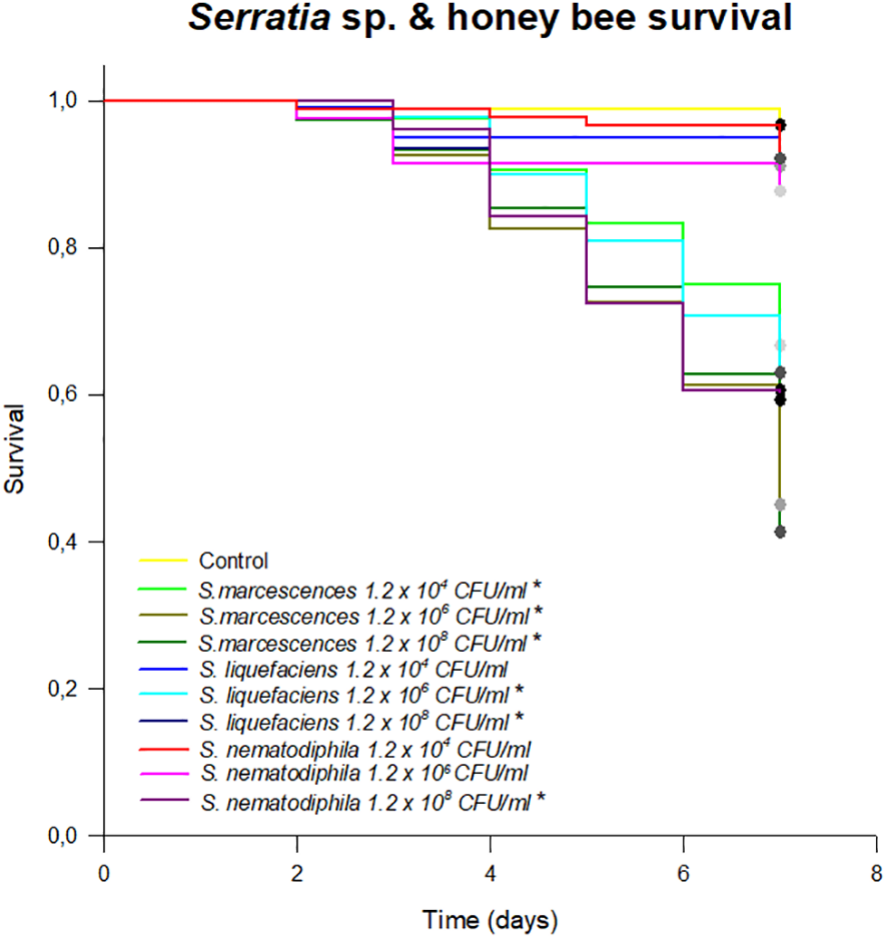
Survival test expressing the % of honeybees survived in an 8-day test following the chronic exposure to different species of *Serratia*. Asterisk (*) indicates statistical differences with the control treatment (syrup without *Serratia*). *p < 0.01.

A total of 18 strains were isolated from the honeybee gut on GYC (**Supplementary Table S1**). Among these, after the 16S rRNA gene sequencing according to Alberoni et al. (2019), three bacterial species were identified as *Serratia*: *S. marcescens* AC8, *S. liquefaciens* AC4, and *S. nematodiphila* 1A (NCBI accession numbers MG649995, MG649996, and MG650059, respectively), and they were used for a survival assay to test their potential pathogenicity and virulence on honeybees. The results of the survival test showed a high pathogenicity of the *S. marcescens* strain when administered through feed. *S. liquefaciens* AC4 and *S. nematodiphila* 1A also caused a statistically significant mortality starting from the concentration of 1.2 × 10^6^ (*p* < 0.01; **Figure 1**). Although a high infective dose in the environment is unlikely to be found, this test confirms that *S. marcescens* may be highly pathogenic when encountered by honeybee. Moreover, a lower infective dose selected such as 10^4^ CFU/mL of sugar syrup led to high mortality, whereas *S. liquefaciens* and *S. nematodiphila* caused a honeybee mortality only at the highest doses (10^6^–10^8^ CFU/mL), confirming the higher pathogenicity of *S. marcescens*.

### 3.2 *Serratia* abundance in the gut correlates with the *Serratia* abundance in the hemocoel

**Figure 2:**
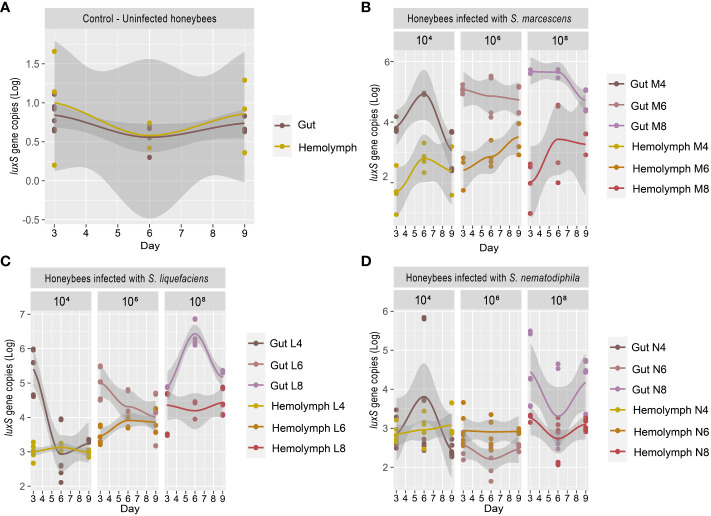
**(A–D)** Experiment 1: *Serratia* absolute quantification in the gut and hemocoel. **(A)** Scatter plot with regression curve of *Serratia luxS* gene copies in both gut and hemocoel in uninfected honeybees [C]. **(B)** Scatter plot with regression curve of *Serratia luxS* gene copies in both gut and hemocoel in honeybees infected with *S. marcescens* AC8 at different infection doses of 10^4^, 10^6^, and 10^8^ [M4, M6, M8]. **(C)** Scatter plot with the regression curve of *Serratia luxS* gene copies in both gut and hemocoel in honeybees infected with *S. liquefaciens* AC4 at different infection doses of 10^4^, 10^6^, and 10^8^ [L4, L6, L8]. **(D)** Scatter plot with regression curve of *Serratia luxS* gene copies in both gut and hemocoel in honeybees infected with *S. nematodiphila* 1A at different infection doses of 10^4^, 10^6^, and 10^8^ [N4, N6, N8]. Sampling times at day 3, day 6, and day 9.

Previous research works (Burritt et al., 2016; Raymann et al., 2018) have shown that particularly virulent strains of *Serratia* can proliferate in the hemocoel and cause a rapid death of the honeybees. Burritt et al. (2016) suggested that the transmission of the pathogen to the hemocoel was triggered by *V. destructor*, which acted as a vector, exactly as it happens for viruses such as Deformed Wing Virus (DWV) and Acute Bee Paralysis Virus (ABPV) (reviewed in Rosenkranz et al., 2010). However, this transmission mechanism may not be the only one (reviewed in Vallet-Gely et al., 2008). *Serratia* could also cross the intestinal epithelial barrier and colonize the hemocoel, and, from there, over-proliferate, causing septicemia. The test described in the present work, in which honeybees were infected with different strains of *Serratia* since the beginning of the test, aims at validating this proliferation path, which might easily escape the treatments for the control of *Varroa* mites. Our work shows that, in honeybee not inoculated with *Serratia*, the basal *Serratia* amount did not vary between the gut and the hemocoel and resulted below the detection limit (1 gene copy) for all the sampling times T1 (3 days), T2 (6 days), and T3 (9 days), respectively (**Figure 2A**). However, following the infection with *S. marcescens* at different concentrations, the number of *S. marcescens* cells in both gut and hemocoel proportionally increased according to the inoculum but did not significantly vary between the sampling times, as shown in **Figure 2B**; **Supplementary Table S2**. Moreover, our results show that the increase of *S. marcescens* load in the gut corresponded to a proportional infection in the hemocoel, during the first 6 days after *Serratia* inoculation. Then, *S. marcescens* counts decreased in the gut, while they increased in the hemocoel. The total number of cells in the hemocoel confirmed that the presence of this pathogen in the gut may act as a reservoir, and it may spread, causing septicemia. *S. liquefaciens* and *S. nematodiphila* strain infections did not follow the same growing trend linked to the inoculation dose (**Figures 2C, D**). The growth and colonization ability of *S. liquefaciens* and *S. nematodiphila* was therefore different from *S. marcescens* (except for the highest dose), and this confirms the lower virulence observed in the survival test. Even when the higher *Serratia* cell number was observed for *S. liquefaciens* after 6 days from inoculation, this development of the bacteria in the gut did not lead to a higher mortality.

Our results therefore confirm that when *Serratia* is present in the honeybee gut, the infection can spread to hemocoel. Furthermore, depending on the initial inoculum, *Serratia* significantly increased the mortality of the host in the first couple of days after infection, with *S. marcescens* as the most virulent of the tested strains. Our results also suggest that *Serratia* can pass through the gut epithelial barrier, and it can colonize the hemocoel where it can also reach higher cell numbers compared to the gut. This dynamic could lead to septicemia, and the number of *Serratia* cells that actually developed in the gut, and then spilled to other insect organs, like hemocoel, remains undetermined. Since *Serratia* can overcome the gut epithelial barrier, the presence of a co-occurring infection of *V. ceranae*, a pathogen able to damage the gut epithelium (Tadei et al., 2020), may facilitate the transfer of *Serratia* in the hemolymph and its proliferation in the hemocoel.

### 3.3 *Serratia* load shows correlation with *V. ceranae* under laboratory conditions

**Figure 3:**
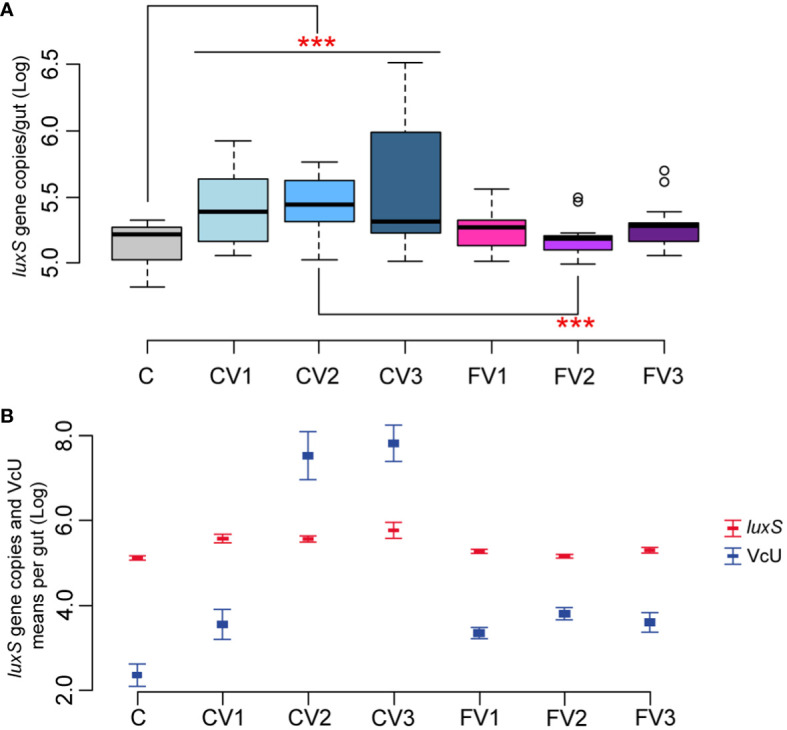
**(A, B)** Experiment 2: *Serratia* absolute quantification—Different doses of *V.* spores. **(A)** Box plots from experiment A showing the Log of *luxS* gene copies/intestine per honeybee gut obtained at 14 days post-inoculation. **(B)** Plot reporting the mean values of *V. ceranae* units (VcU) from Garrido et al. (2024) and *S. marcescens* (*luxS*) for the different experimental conditions. Bees were inoculated with 5×10^2^ spores of *V. ceranae* [CV1], 5×10^3^ spores of *V. ceranae* [CV2], 5×10^4^ spores of *V. ceranae* [CV3], 5×10^2^ spores of *V. ceranae* and Fumagillin [FV1], 5×10^3^ spores of *V. ceranae* and fumagillin [FV2], and 5×10^4^ spores of *V. ceranae* and Fumagillin [FV3]. [C] represents the non-infected control. ***p < 0.01.

The effect of different *V. ceranae* spore loads on *Serratia* naturally infected honeybees was therefore investigated (**Table 1**, Section 2). Honeybees infected with different spore doses showed a significant increase in *Serratia* absolute abundance when compared to uninfected control (*p* < 0.01 for C vs. CV1, CV2 and CV3), regardless of the number of inoculated spores. In contrast, fumagillin-treated honeybees infected with different *V. ceranae* doses did not show a significant increase in *Serratia* (C vs. FV1, FV2, and FV3) (**Figure 3A**). In addition to these results, our findings highlight a positive correlation between *Vairimorpha* infection (expressed as *V. ceranae* units—VcU) and the level of *Serratia* (correlation +0.81, *p* < 0.01), suggesting that the epithelial gut disruption by the *Vairimorpha* parasite contributes to this pathogen spread (**Figure 3B**). However, further studies are required to confirm this interaction among the two pathogens.

*Serratia* did not proliferate when the antibiotic fumagillin was supplied, confirming that the control of *V. ceranae* infection also limits the proliferation of *Serratia*, contributing to improving honeybee health. Fumagillin is known to have an anti-amebic and anti-microsporidia action, but no anti-bacterial activity. This was confirmed in our work with an assay targeted to detect any possible antimicrobial activity of fumagillin, at different doses, on the three isolated *Serratia* strains. Fumagillin did not show any direct antimicrobial activity against *Serratia in vitro* in a well diffusion assay, at all tested concentrations (see Section 2.4), suggesting an indirect action of the antimycotic agent on *Serratia* proliferation. This effect is particularly evident in the honeybees infected with 5×10^3^ *Vairimorpha* spores and treated with fumagillin (**Figures 3A, B**) where the comparison CV2 vs. FV2 was found significant (*p* < 0.01), but the reduction, although not significant, is also present in honeybees infected with 5×10^2^ and 5×10^4^ *Vairimorpha* spores and treated with the antibiotic fumagillin (FV1 and FV3).

### 3.4 *Serratia* load in the presence of different microbial treatments (single strains and bacterial mixture)

**Figure 4:**
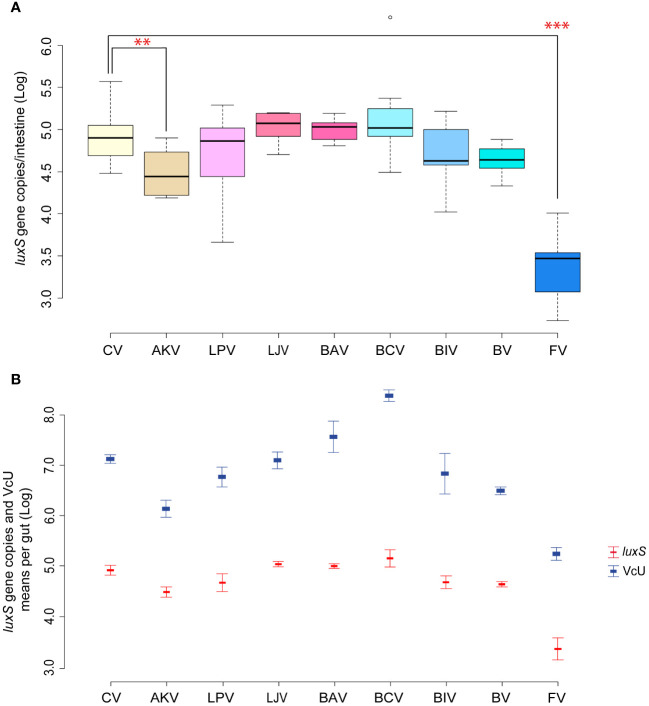
**(A, B)** Experiment 3: *Serratia* inhibition—Single strains. **(A)** Box plots from experiment 3a reporting the Log of *luxS* gene copies*/*intestine per honeybee gut. **(B)** Plot reporting the mean values of *V. ceranae* units (VcU) from Garrido et al. (2024) and *S. marcescens* (*luxS*) for the different experimental conditions. Honeybees were inoculated with 5×10^4^ spores of *V. ceranae* (marked with “N”) for every treatment with the administration of sugar syrup (1:1 w/v) mixed with the following dietary ingredients: [CV] infected control, [AKV] *A. kunkeei*, [LPV] *L. plantarum*, [LJV] *L. johnsonii*, [BAV] *B. asteroides*, [BCV] *B. coryneforme*, [BIV] *B. indicum*, [BV] Bacteria Mixture, and [FV] fumagillin. **p < 0.05; ***p < 0.01.

In Garrido et al. (2024), the oral administration of the single bacterial strains composing a previously selected beneficial bacterial mixture aimed at controlling *V. ceranae* showed that only *A. kunkeei* significantly reduced the load of the parasite in the gut. In the present work, similar results were obtained in the reduction of *Serratia*, whose absolute abundance was significantly reduced in the samples in which *A. kunkeei* was administered (*p* < 0.05) (*Serratia* counts from 4.92 [CV] to 4.52 [AKV] Log *luxS* gene copies/gut; **Figure 4A**). *Serratia* amount in all samples has the same trend of *V. ceranae* counts previously reported (**Figure 4B**), showing a positive Pearson correlation of + 0.89 (*p* < 0.01). Given the properties characterizing several *Lactobacillaceae* members, the inhibition of *Serratia* colonization could be due to the production of antimicrobial peptides (Forsgren et al., 2010; Palmer-Young et al., 2019) and/or acidification of the gut lumen. On the other hand, it is interesting to note that the administration of the bacterial mixture, although not statistically significant, led to a reduction of *Serratia* as obtained for *Vairimorpha*.

As reported by Olofsson et al. (2016) and Kačániová et al. (2019), different strains of *A. kunkeii* such as Fhon2, Anhmro10, and Yubipro16 possess a high antimicrobial activity *in vitro* against *S. marcescens*. Furthermore, the tendency to reduce the infection of *Serratia* when *B. indicum* DSM 20214 or *L. plantarum* Dan91 are administered is evident, although not statistically significant. It is interesting to note that the two best-performing strains against *Serratia* are exogenous to the honeybee gut. The first one (*A. kunkeei*) is an environmental microorganism that is usually found in flowers and in honeybee and is usually limited to the crop. The second (*B. indicum*) is a typical commensal microorganism of *Apis cerana* (Asian honeybee) where it was isolated for the first time. It is therefore plausible that these two microorganisms triggered an immune response in the tested honeybee, as was shown by Garrido et al. (2024). Indeed, it was possible to correlate specific responses to the pathogen with target microbial strains (Steele et al., 2017; Horak et al., 2020). On the other hand, according to our results, the use of two native bifidobacteria species (*B. asteroides* and *B. coryneforme*) increases both *Serratia* and *V. ceranae* counts. This is in line with recent research showing a strict connection between *V. ceranae* and *Bifidobacterium* increase in the gut lumen (Zhang et al., 2019), probably due to a triggered gut dysbiosis, which might explain the synergy between some bifidobacteria, *Nosemosis*, and *Serratia* negatively affecting honeybee health.

## 4 Conclusion

Among the different *Serratia* species isolated from honeybee gut, the *S. marcescens* strain was the most virulent on honeybees compared to the *S. liquefaciens* and *S. nemathodiphila* species. The results obtained allowed us to conclude that *S. marcescens* can overcome the epithelial gut barrier, spread to the hemocoel, and proliferate in the hemolymph. The presence of an active *Vairimorpha* infection that disrupts gut epithelial cells also facilitates the *Serratia* development. Under cage conditions, a strong correlation between *V. ceranae* infection and *Serratia* development has been highlighted. The presence of fumagillin, active against *Vairimorpha* but with no direct activity against *Serratia*, seems to control the development of both pathogens, as well as some bacterial strain supplied as feed additive active against *Vairimorpha.* The microbial strains active against *Vairimorpha*, such as *A. kunkeii*, can reduce also *Serratia*, confirming the correlation between the two pathogens. The detection of a connection between these two chronic pathogens in certain circumstances is of concern for the researcher working under laboratory conditions on honeybees. However, field conditions may mitigate or enhance the pathogens’ spread and synergy in the honeybee colonies. Further investigations are required in order to better understand these two pathogens’ dynamics considering environmental variables as well.

## Data availability statement

The datasets presented in this study can be found in online repositories. The names of the repository/repositories and accession number(s) can be found in the article/**Supplementary Material**. Raw data can be found in Mendeley Data repository at the following DOI: 10.17632/mjc2xky9s7.2.

## Ethics statement

Ethical approval was not required for the studies involving animals in accordance with the local legislation and institutional requirements because according to the Italian and Argentinian law, ethical approval is not necessary for insects. Written informed consent was obtained from the owners for the participation of their animals in this study.

## Author contributions

CB: Formal analysis, Investigation, Methodology, Validation, Writing – original draft. DA: Data curation, Investigation, Writing – original draft, Conceptualization, Formal analysis, Supervision, Validation, Visualization. PG: Funding acquisition, Investigation, Methodology, Writing – review & editing. MP: Funding acquisition, Investigation, Methodology, Writing – review & editing. LB: Data curation, Resources, Visualization, Writing – review & editing. DM: Funding acquisition, Writing – review & editing. DS: Conceptualization, Funding acquisition, Methodology, Writing – review & editing. ME: Funding acquisition, Supervision, Writing – review & editing. DD: Funding acquisition, Project administration, Validation, Writing – original draft.
