# Supplementary material for: Vairimorpha (Nosema) ceranae can promote Serratia development in honeybee gut: an underrated threat for bees?
Source: Front Cell Infect Microbiol. 2024 May 13;14:1323157. doi: 10.3389/fcimb.2024.1323157 (PMC11131372; doi:10.3389/fcimb.2024.1323157)
Supplement: Supplementary file 1 [file DataSheet_1.docx]

Supplementary Material

**Supplementary Materials**

**Table S1** Microbial isolates on GYC identified with the sequencing of the 16S rRNA gene.

| **Accession Number** | **Isolate** | **Identification** |
| --- | --- | --- |
| MG6499954 | AC1 | *Hafnia alvei* |
| Not deposited | AC2 | *Serratia marcescences* |
| Not deposited | AC3 | *Serratia marcescences* |
| MG649995 | AC4 | *Serratia liquefaciens* |
| Not deposited | AC5 | *Serratia marcescences* |
| Not deposited | AC6 | *Obesumbacterium proteus* |
| Not deposited | AC7 | *Serratia liquefaciens* |
| MG649996 | AC8 | *Serratia marcescences* |
| MG649997 | AC9 | *Obesumbacterium proteus* |
| MG649998 | AC10 | *Obesumbacterium proteus* |
| MG650059 | 1A | *Serratia nematodiphila* |
| MG650060 | F2 | *Bacillus licheniformis* |
| MG650058 | F3 | *Paenibacillus yonginensis* |
| MG649992 | XB1 | *Arthrobacter oryzae* |
| MG649990 | CE1 | *Bacillus cereus* |
| MG650020 | Q1Q | *Bacillus toyonensis* |
| MG649989 | CF1 | *Macrococcus equipercicus* |
| MG650021 | T1T | *Microbacterium azadirachtae* |
| MG649988 | PA1 | *Macrococcus yunnanensis* |
| MG649991 | CD1 | *Staphylococcus hominis* |

**Table S2:** Average Log of *luxS* gene copies per honeybee in the different experimental conditions, sampling time and tissue analyzed.

| **Experimental conditions** | **Log of *Serratia* *luxS* gene copies** | | | | | |
| --- | --- | --- | --- | --- | --- | --- |
|  | **Honeybee Gut** | | | **Honeybee Hemolymph** | | |
|  | *T1* | *T2* | *T3* | *T1* | *T2* | *T3* |
| **C** | 0 | 0 | 0 | 1 | 0 | 0 |
| **M8** | 6.00 | 6.08 | 5.85 | 4.27 | 2.04 | 3.54 |
| **M6** | 5.42 | 5.28 | 5.32 | 3.49 | 2.73 | 3.82 |
| **M4** | 4.52 | 3.59 | 3.2 | 3.25 | 1.82 | 2.03 |
| **L8** | 4.90 | 6.43 | 5.18 | 4.36 | 4.20 | 4.43 |
| **L6** | 5.02 | 4.31 | 4.01 | 3.46 | 3.91 | 3.86 |
| **L4** | 5.40 | 2.93 | 3.24 | 3.00 | 3.13 | 2.98 |
| **N8** | 4.44 | 3.31 | 4.17 | 3.27 | 2.73 | 3.10 |
| **N6** | 3.88 | 2.21 | 2.46 | 2.94 | 2.91 | 2.92 |
| **N4** | 2.85 | 4.01 | 2.42 | 2.86 | 2.93 | 3.08 |
